# First-line dose-dense chemotherapy with docetaxel, cisplatin, folinic acid and 5-fluorouracil (DCF) plus panitumumab in patients with locally advanced or metastatic cancer of the stomach or gastroesophageal junction: final results and biomarker analysis from an Italian oncology group for clinical research (GOIRC) phase II study

**DOI:** 10.18632/oncotarget.22909

**Published:** 2017-12-04

**Authors:** Gianluca Tomasello, Nicola Valeri, Michele Ghidini, Elizabeth C. Smyth, Wanda Liguigli, Laura Toppo, Rodolfo Mattioli, Alessandra Curti, Jens C. Hahne, Federica M. Negri, Stefano Panni, Margherita Ratti, Silvia Lazzarelli, Fabiana Gerevini, Chiara Colombi, Andrea Panni, Massimo Rovatti, Leonardo Treccani, Mario Martinotti, Rodolfo Passalacqua

**Affiliations:** ^1^ Oncology Division, ASST Ospedale di Cremona, Cremona, Italy; ^2^ Division of Molecular Pathology, The Institute for Cancer Research, Sutton, London, UK; ^3^ Department of Medicine, The Royal Marsden Hospital, Sutton, London, UK; ^4^ Oncology Division, Ospedali Riuniti Marche Nord, Fano, Italy; ^5^ Pathology Division, ASST Ospedale di Cremona, Cremona, Italy; ^6^ Surgery Division, ASST Ospedale di Cremona, Cremona, Italy; ^7^ Radiology Division, ASST Ospedale di Cremona, Cremona, Italy

**Keywords:** gastric cancer, DCF, panitumumab, dose-dense, chemotherapy

## Abstract

**Background:**

Survival for patients with advanced gastroesophageal cancer (AGC) using standard treatment regimens is poor. EGFR overexpression is common in AGC and associated with poor prognosis. We hypothesized that increasing the dose intensity of chemotherapy and adding panitumumab could improve efficacy.

**Methods:**

HER2 negative, PS 0-1 patients, received up to 4 cycles of panitumumab 6 mg/kg d 1, docetaxel 60 mg/m2 d 1, cisplatin 50 mg/m2 d 1, l-folinic acid 100 mg/m2 d 1-2, followed by 5-FU 400 mg/m2 bolus d 1-2, and then 600 mg/m2 as a 22 h c.i. on d 1-2, q15 d, plus pegfilgrastim 6 mg on d 3. Patients with disease control after 4 cycles received panitumumab until progression.

**Results:**

From 05/2010 to 01/2014, 52 patients (75% male; median age 64.5 y; metastatic 90%, locally advanced 10%; 96% adenocarcinoma; 25% GEJ) were recruited. Three CR, 29 PR, 10 SD and 8 PD were observed, for an ORR by ITT (primary endpoint) of 62% (95% CI, 48%-75%) and a DCR of 81%. Median TTP was 4.9 months (95% CI, 4.2-7.0) and mOS 10 months (95% CI, 8.2- 13.5). Most frequent G3-4 toxicities: leucopenia (29%), asthenia (27%), skin rash (25%), neutropenia (19%), anorexia (17%), febrile neutropenia (13%), and diarrhea (15%). EGFR expression tested both with dd-PCR and FISH was not associated with any significant clinical benefit from treatment.

**Conclusions:**

Dose-dense DCF plus panitumumab is an active regimen. However, the toxicity profile of this limits further development. Further research on predictive biomarkers for treatment efficacy in AGC is required.

Clinical trial information: 2009-016962-10.

## INTRODUCTION

Gastroesophageal cancer is a globally important disease; together, gastric and esophageal cancer are responsible for more than 1.1 million deaths annually [1]. Surgical resection represents the only curative treatment option; however, even for patients who undergo potentially curative surgery in conjunction with neoadjuvant or adjuvant therapy, relapse is common [2-5]. Additionally, in countries without screening programs, most patients present with unresectable or metastatic disease and are treated with systemic chemotherapy with palliative intent. Patients with advanced gastroesophageal cancer have a median survival in clinical trials of first line chemotherapy of less than one year; therefore, improved treatment options are desirable for these patients [6, 7].

Standard chemotherapy for patients with advanced gastroesophageal cancer is a cisplatin and fluoropyrimidine doublet, with the addition of either a taxane or anthracycline for fit patients [6, 7]. One of the more active schedules is the combination of docetaxel, cisplatin and 5-FU (DCF), which in a randomized phase III trial, was associated with a significant progression free and overall survival benefit, achieved at the cost of increased toxicity [7]. One modified DCF regimen is a two-week interval (“dose-dense”) schedule, with the support of granulocyte colony-stimulating factors (G-CSFs). We recently reported on the feasibility and activity of this regimen, and demonstrated it to be safe, and associated with a 61% objective response rate [8].

Epidermal growth factor receptor (EGFR) pathway dysregulation is present in a variety of solid tumors. In gastroesophageal cancer, EGFR overexpression is frequently observed and associated with an unfavourable prognosis [9]. Panitumumab is a high affinity human IgG2 monoclonal antibody directed against human EGFR which blocks the binding of the ligands EGF, TGFα, amphiregulin, betaregulin, epiregulin, and heparin-binding EGF [10]. Panitumumab has demonstrated efficacy in RAS wild type metastatic colorectal cancer (mCRC) [11]. In gastroesophageal cancer, the frequency of KRAS mutations is low in comparison to mCRC, and therefore anti-EGFR therapy could be effective for a larger proportion of patients [12-14]. We hypothesized that the addition of panitumumab to dose-dense DCF could improve outcomes for patients with advanced gastroesophageal cancer (AGC) and evaluated this assumption in a clinical trial.

## RESULTS

### Patients

Fifty-two consecutive patients from 2 Italian oncology centers were enrolled from May 2010 to January 2014. Patients’ demographics and disease characteristics are reported in Table 1. Ninety percent of patients were metastatic and 10% had locally advanced and unresectable cancers. Ninety-six percent were assessable for response and all for toxicity. Patient characteristics were consistent with those in other clinical trials of advanced gastroesophageal cancer. The median age of patients enrolled was 64.5 years and half of the patients were >65 years old. Thirty-nine (75%) patients were male. Thirty-one percent of patients had more than one site of metastatic disease. Almost all the patients had adenocarcinoma as histology, one patient had a poorly differentiated carcinoma, and another invasive carcinoma intestinal type.

**Table 1:** Patients’ demographics and disease characteristics

	N	%
**Enrolled Patients**	52	100
**Metastatic**	47	90
**Locally advanced not resectable**	5	10
**Assessable for toxicity**	52	100
**Assessable for response**	50	96
**Age**	64.5 years (median) 26 pts aged >65 years	range 42-75 years 50
**Sex**		
**Male**	39	75
**Female**	13	25
**Performance Status:**		
**0**	27	52
**1**	25	48
**Tumor location**		
**Stomach**	39	75
**Gastroesophageal junction**	13	25
**Metastatic sites: lung**	9	17
**lymphnodes**	40	77
**bone**	6	11
**liver**	21	40
**peritoneum**	21	40
**other**	6	11
**>1 metastatic site**	31	60
**Histology:**		
**ADK**	50	96
**Other**	2	4
**Tumor grade**		
**1**	1	2
**2**	12	23
**3**	39	75

### Efficacy and safety

**Figure 1:**
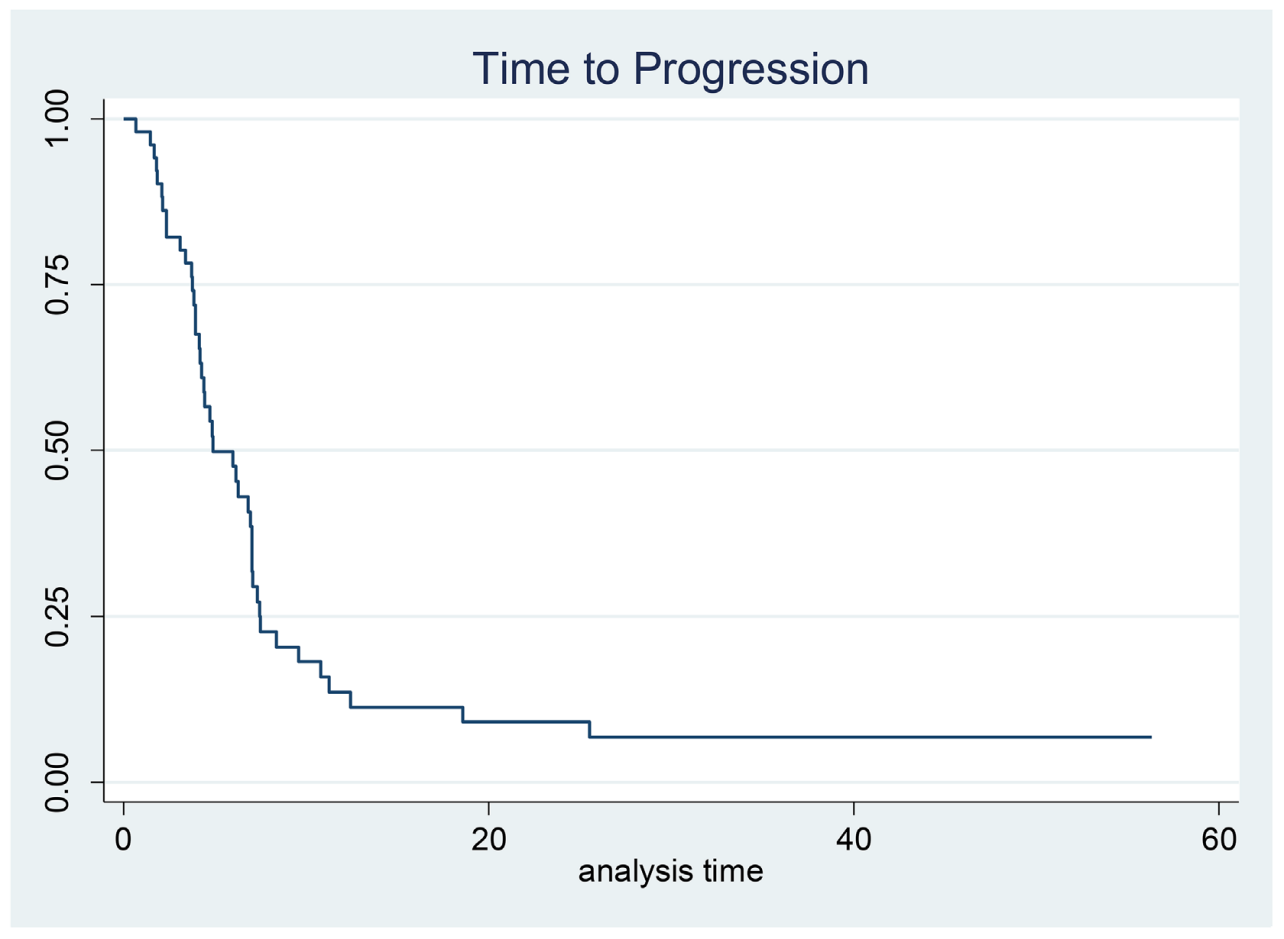
Kaplan–Meier estimates for time to progression

**Figure 2:**
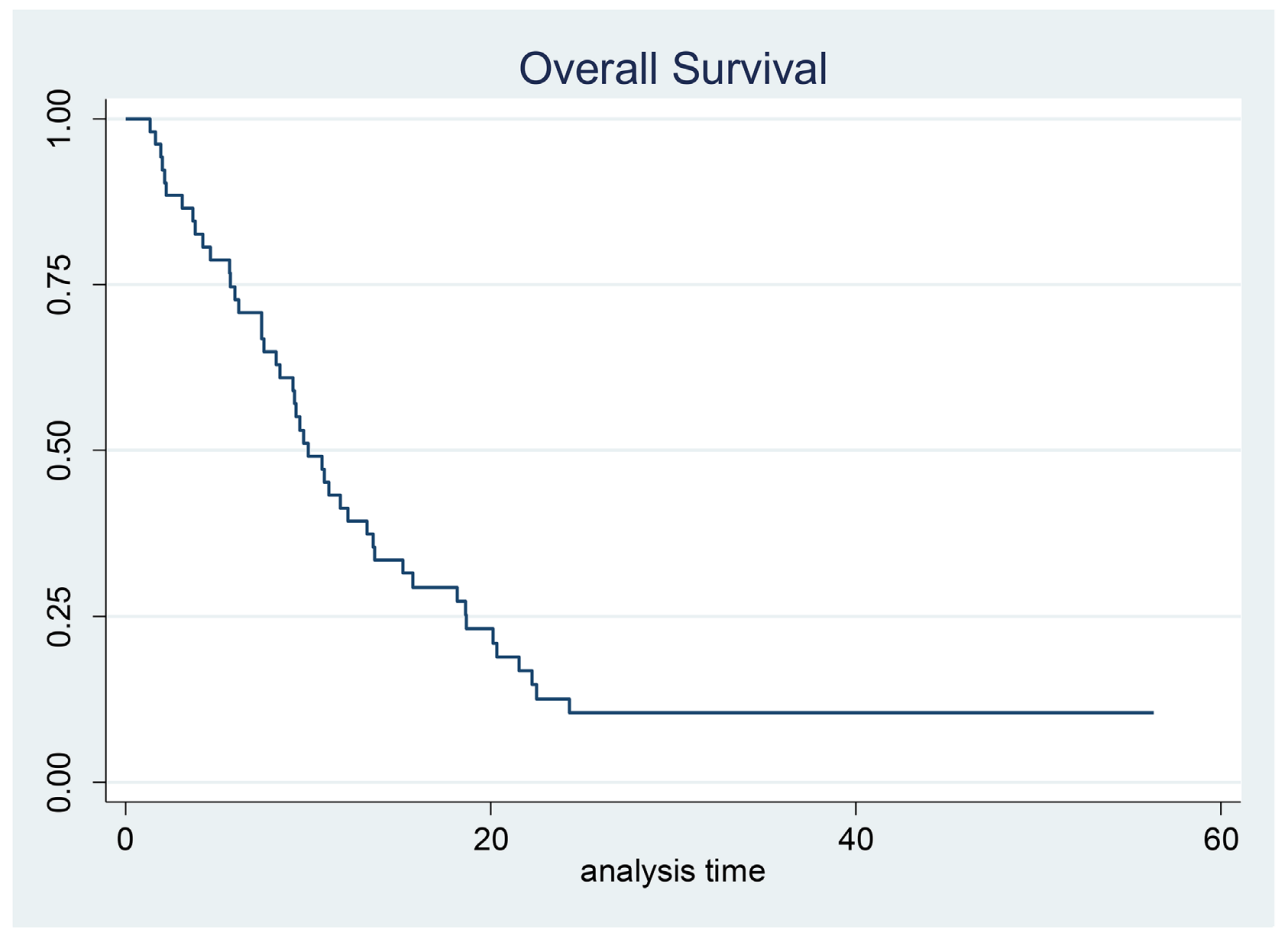
Kaplan–Meier estimates for overall survival

A median of four cycles (range, 1-6) per patient were administered. Less than half of patients (22/52, 42%) completed treatment without any dose reduction or delay. Fifty (96%) patients were evaluable for response: one patient died after 3 cycles of chemotherapy due to myocardial infarction and another died after the first cycle due to bowel obstruction, neither were believed by the investigator to be related to study treatment. All patients were available for toxicity assessment. Three patients had a CR and 29 had a PR, corresponding to an ORR of 64% in the evaluable for response population (95% confidence interval (CI), 51.0–77.0%) and 62% (95% CI,48.0-75.0%) in the ITT population. Stable disease (SD) was reported in 10 patients (20%) and progressive disease (PD) in 8 patients (16%); thus, the clinical benefit rate was 84% for the evaluable for response population (Table 2). Objective response rates in patients >65 years were lower than in younger patients (46% vs 77%, respectively). Twenty-six (50%) patients entered the maintenance phase with single agent panitumumab and received a median of 7.3 cycles (range 1.0-46.0). After a median follow-up of 33.6 months (95% CI, 24.8-[) median TTP was 4.9 months (95% CI, 4.2-7.0) and median OS was 10.0 months (95% CI, 8.2- 13.5) (Figures 1 and 2). Median OS in patients >65 years was 8.4 months (95%CI 5.6-11.7). Seven patients (3 locally advanced and 3 metastatic) not receiving maintenance therapy underwent gastrectomy. In an exploratory analysis comparing this subgroup with patients with no surgery (24) or gastrectomized before study entry (21), median OS was 20.0, 8.4 and 9.7 months, respectively.

**Table 2:** Efficacy: Intention-to-treat analysis

Response	N	%
**Partial Response**	29	56
**Complete Response**	3	6
**Stable Disease**	10	19
**Progression**	8	15
**ORR**	32	62
**DCR**	42	81
**Not evaluable**	2	4

Toxicities observed during treatment are listed in Table 3. The most frequent G3-4 toxicities were: leucopenia (29%), neutropenia (19%), febrile neutropenia (13%), anemia (10%), asthenia (27%), mucositis (13%), anorexia (17%), nausea/vomiting (12%), diarrhea (15%), hypokalemia (12%), and skin rash (25%). Two toxic deaths were registered (pulmonary aspergyllosis due to febrile neutropenia and gastric hemorrhage). Severity of dermatologic toxicity after first cycle was significantly correlated with response to treatment (p=0.0134), but not with OS (p=0.1324).

**Table 3:** Toxicity according to NCI CTC version 3.0 criteria

Grade 3/4 toxicity	N=52	%
**Leucopenia**	15	29%
**Neutropenia**	10	19%
**Febrile Neutropenia**	7	13%
**Anemia**	5	10%
**Thrombocytopenia**	4	8%
**Asthenia**	14	27%
**Mucositis**	7	13%
**Nausea/Vomiting**	6	12%
**Diarrhea**	8	15%
**Skin toxicity**	13	25%
**Hypomagnesemia**	2	4%
**Toxic deaths**	2	4%

### Molecular analysis

**Figure 3:**
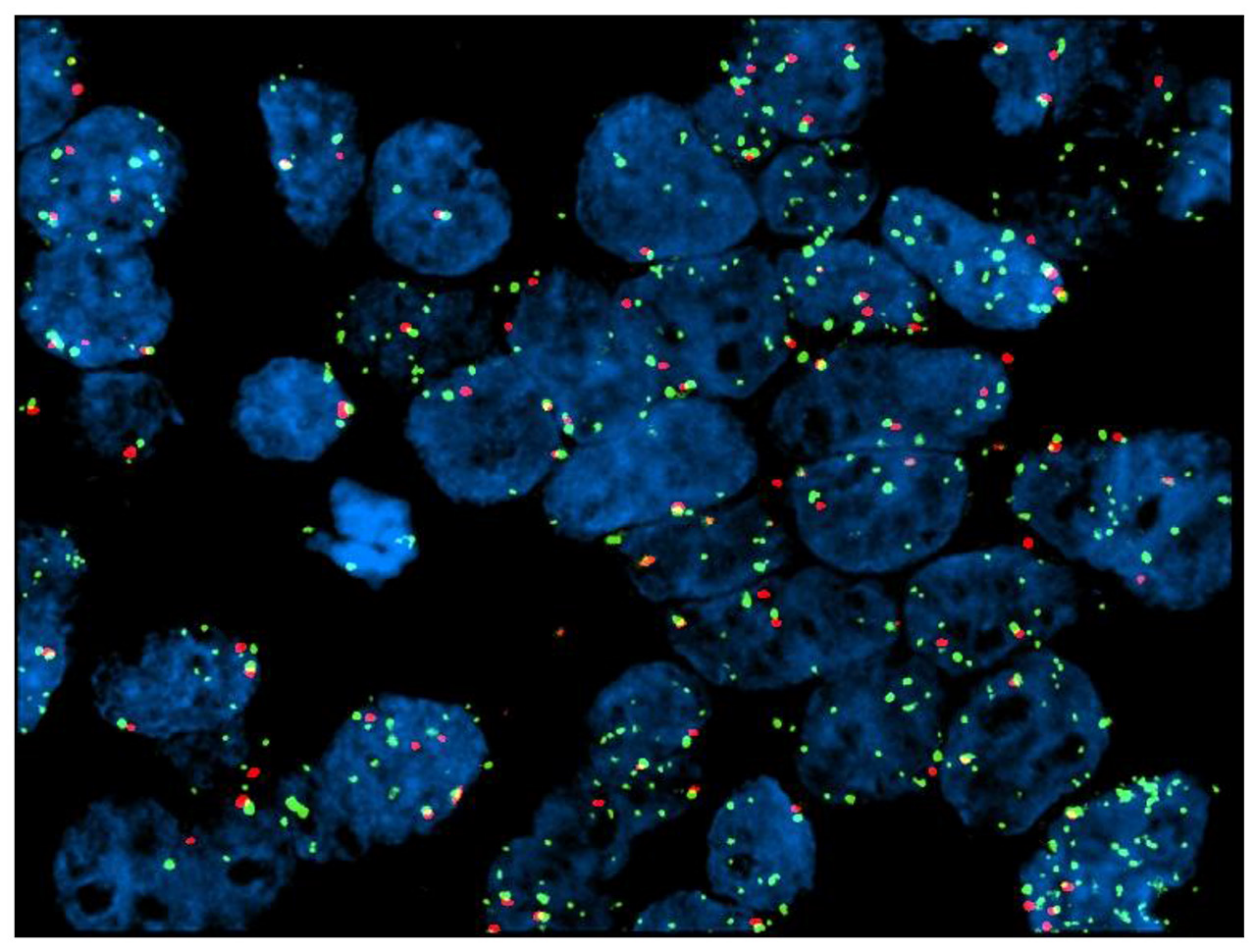
Case no. 32a (cerebellar metastasis from GC): FISH test shows partial amplification

**Figure 4:**
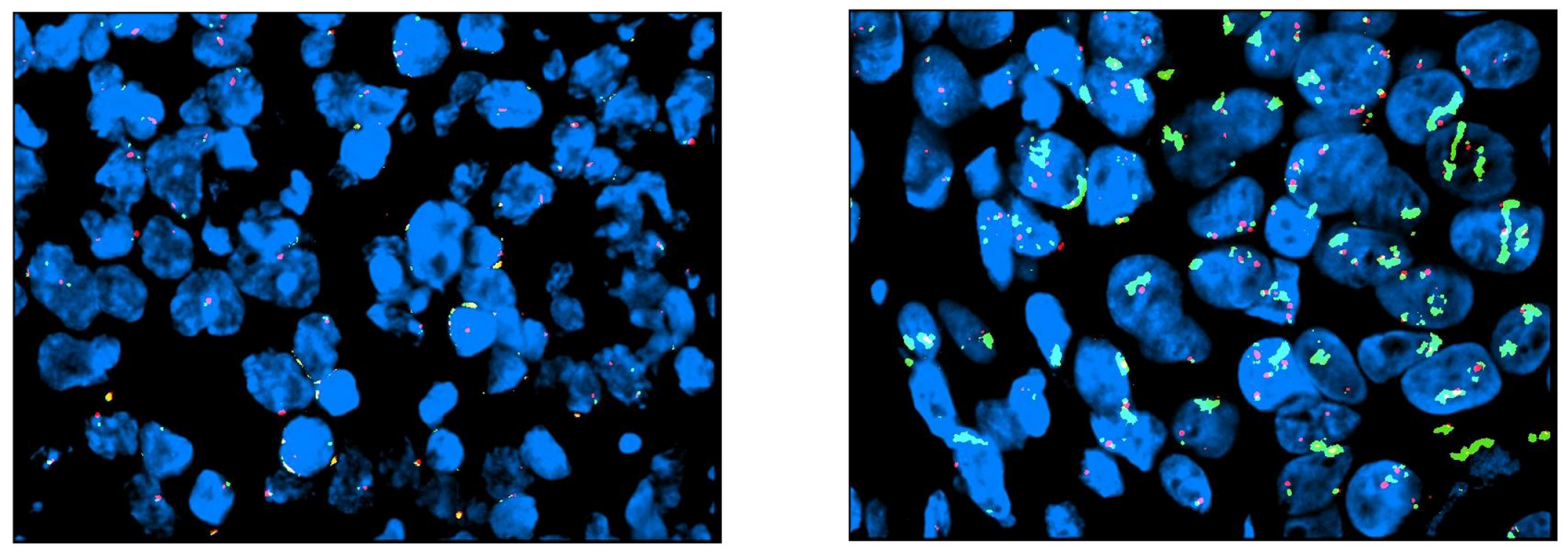
Case no. 24 (biopsy from primary GC): FISH shows partial amplification in a single cell cluster (left)

DdPCR analysis was performed on 48 tissue samples derived from 45 patients. In the remaining seven cases, the amount of tumor cells available was insufficient for the planned analysis. Three patients had tissue available from more than one site. Table 4 demonstrates the results for ddPCR CNV-assay for EGFR. Five out of 45 patients (11%) demonstrated EGFR copy number gain using ddPCR. In the first case (1a, gastric biopsy, Table 4), ddPCR analysis reported a EGFR CNV of 10, while the correspondent resection sample (1b, Table 4) showed no EGFR amplification. FISH analysis was negative for amplification in both cases. Sample 9a (gastric biopsy, Table 4) harbored a EGFR CNV of 5, while the liver biopsy sample had no amplification. FISH confirmatory analysis gave a negative result. In the third case, cerebellar biopsy resulted in high EGFR CNV (52.5 CNV, case 32a, Table 4), while the liver biopsy sample had no amplification reported (case 32b, Table 4). FISH analysis was strongly positive for amplification only in case 32a (Table 4, Figure 3). DdPCR analysis reported high values of CNV in two further cases of gastric biopsies (cases 18 and 24, Table 4). FISH was positive for EGFR amplification in case 18, while partial amplification in a single cell cluster was recorded for case 24 (Figure 4). Out of these 5 ddPCR amplified patients, 2 had a partial response (case 1 and 9). However, such responses were of short duration and were associated with survival times below the median overall registered.

**Table 4:** Molecular analyses: results for ddPCR CNV-assay for EGFR and confirmatory FISH test

	ddPCR			FISH
Patient number	Measurement with 50 ng DNA	Repeated measurement 1	Repeated measurement 2	FISH amplification
1a (gastric biopsy)	10	n.d.	n.d	not amplified
1b (gastric resection)	1,07	n.d.	1,8	not amplified
2	1,15	1,09	n.d.	
3	2,01	n.d.	3,17	
4	n.d.	n.d.	1,3	
5	1,5	n.d.	2,19	
6	2	n.d.	1,1	
7	1,1	0,6	1,7	
8	1,01	n.d.	1,44	
9a (gastric biopsy)	5	n.d.	n.d.	not amplified
9b (liver biopsy)	0,41	n.d.	2,03	not amplified
10	0,36	n.d.	0,8	
11	1,2	1,3	n.d.	
12	0,4	n.d.	2,4	
13	1,5	n.d.	1,5	
14	0,58	n.d.	1,84	
15	n.d.	2,4	1,5	
16	0,63	n.d.	1,4	
17	n.d.	1,14	1,8	
18	3,26	n.d.	4,77	amplified
19	0,12	n.d.	2,2	
20	0,59	0,68	n.d.	
21	1,4	2	n.d.	
22	n.d.	1,31	1,13	
23	0,56	2	0,7	
24	1,21	n.d.	5,5	partial amplification (one cluster)
25	n.d.	0,5	2,3	
26	1,4	n.d.	n.d.	
27	n.d.	2,5	2,3	
28	0,96	n.d.	1,08	
29	0,92	n.d.	1,06	
30	0,73	n.d.	1,72	
31	n.d.	0,64	0,79	
32a (cerebellum biopsy)	23,5	n.d.	52,5	amplified
32b (liver biopsy)	0,47	n.d.	1,41	not amplified
33	0,42	n.d.	1,8	
34	0,33	n.d.	1,2	
35	0,54	n.d.	0,85	
36	1,25	n.d.	3,6	
37	0,93	n.d.	1,04	
38	0,82	n.d.	2	
39	n.d.	1,04	n.d.	
40	n.d.	1,33	1,27	
41	n.d.	n.d.	n.d.	
42	n.d.	1,3	n.d.	
43	n.d.	1,92	1,8	
44	n.d.	0,95	0,89	
45	n.d.	2,02	2,66	

## DISCUSSION

In this study, we evaluated the efficacy of the addition of panitumumab to dose-dense modified DCF therapy for patients with AGC. To our knowledge, this is the first study evaluating three potentially important variables in AGC: a dose-dense chemotherapy triplet; the combination with an anti-EGFR monoclonal antibody and a maintenance therapy with panitumumab. We found that although dose-dense DCF plus panitumumab was associated with an encouraging response rate of 64% (meeting the primary endpoint for the study), this was not significantly improved from the same regimen without the addition of panitumumab [8] and that toxicity was significant. However, this increased toxicity did not have a detrimental effect on OS in our study as TTP and OS are in line with the literature.

Whilst this study was recruiting, two large randomized trials reported on the efficacy of combining an anti-EGFR antibody to cytotoxic chemotherapy in patients with AGC. Both the EXPAND and the REAL3 study failed to demonstrate any significant survival benefit from the addition of cetuximab or panitumumab to standard cytotoxic chemotherapy, respectively [16, 17]. The REAL3 trial randomly assigned 553 patients with previously untreated advanced unselected esophagogastric cancer to EOX (epirubicin 50 mg/m^2^ on day 1, oxaliplatin 130 mg/m^2^ on day 1, and capecitabine 1250 mg/m^2^ per day), or modified EOC (with a reduction in oxaliplatin to 100 mg/m^2^ and capecitabine to 1000 mg/m^2^ per day) plus panitumumab. The addition of panitumumab produced similar response rates, but was associated with a significantly worse OS (median 8.8 versus 11.3 months). However, in the REAL-3 study, doses of oxaliplatin and capecitabine were reduced in the experimental arm compared to standard EOX due to increased rates of diarrhea when EOX was combined with panitumumab. In contrast, when cetuximab was used in conjunction with standard dose cisplatin and capecitabine in the EXPAND trial, no detriment nor benefit was seen from the addition of anti-EGFR therapy. Based upon these disappointing results, further trials with anti-EGFR therapy have not been pursued in AGC.

As occurred in the REAL-3 study, the toxicity associated with dd-DCF-P was not negligible. Only 42% of patients managed to complete the dose-dense schedule with no dose reductions. Compared with toxicity registered in our previous study of only chemotherapy [8], the addition of panitumumab to dd-DCF (at slightly lower doses) led to a clinically meaningful increase of specific adverse events such as febrile neutropenia (13% vs 6%) and diarrhea (15% vs 4%). The prophylactic use of G-CSFs made it possible to limit febrile neutropenia cases to less than 15%, but intense fatigue was particularly frequent and demonstrated in more than 25% of patients. As expected, high grade skin rash was typically associated with panitumumab use and occurred in one in four subjects. However, no patient received any pre-emptive antibiotic treatment for skin rash. Despite receiving a 30% dose reduction upfront, 7 out of 26 patients aged > 65 years (27%) had further dose reductions. In patients aged <65 years dose reduction occurred in 65% of cases (17/26). Although median TTP and OS did not appear compromised with dd-DCF-P compared to historical controls, these tolerability issues limit further development.

In previous studies addressing the value of anti-EGFR therapy in AGC, patients were not biomarker selected. However, in the EXPAND study a post-hoc analysis demonstrated no difference in survival according to EGFR immunohistochemistry score (with a median score of 0) [16]. Similarly, in our post-hoc molecular analysis performed with ddPCR on available tissue samples, we did not find any correlation between EGFR CNV and clinical benefit from dd-DCF-P. Specifically, only 2 out of 5 patients with ddPCR amplified showed an objective response to treatment. Unfortunately, such responses were of short duration and did not translate into any significant overall survival prolongation. However, as the proportion of patients with EGFR copy number gain in our dataset is small, these results cannot be considered definitive. Our findings are consistent with the proportion of advanced gastroesophageal cancer patients described as having EGFR amplification in the literature; 5/45 (11%) of patients demonstrated EGFR copy number gain using ddPCR, a proportion which is consistent with that reported in the gastric cancer TCGA [18]. Our results also highlight the issue of heterogeneity of biomarker expression in AGC; discordant EGFR CNV results were demonstrated between two different metastatic sites from the same primary (case 32 a and b, Table 4 and Figure 3), whereas in another ddPCR amplified case only a partial EGFR amplification limited to a cell cluster was noted (case 24, Table 4 and Figure 4). Similar findings demonstrating intra-tumor and intra-sample heterogeneity of receptor tyrosine kinase amplification (including HER2, EGFR and FGFR2) expression in gastric cancer, have previously been reported [19-22]. Intra-tumoral heterogeneity of biomarker expression in gastroesophageal cancer has significant implications for the success of targeted therapy delivery for this patient population [20, 23, 24].

In conclusion, although dose-dense DCF chemotherapy combined with panitumumab was an active regimen in an unselected population of patients with AGC, significant safety issues considerably limit any further clinical development of this regimen. Molecular heterogeneity in gastric cancer may be one of the reasons rendering the identification of reliable predictive biomarkers for targeted therapies difficult to obtain. In future, increasing use of liquid biopsies, which offer a complete picture of tumor heterogeneity, will probably represent a key avenue for better exploiting the potential of targeted agents in AGC patients.

## MATERIALS AND METHODS

### Trial design

This was a phase II single-arm multicenter trial of dose-dense chemotherapy with docetaxel, cisplatin, l-folinic acid and 5-fluorouracil (DCF) plus panitumumab in patients with locally advanced or metastatic cancer of the stomach or gastroesophageal junction (GEJ).

### Participants

Inclusion criteria were as follows: histologically confirmed metastatic carcinoma of the stomach or GEJ or locally advanced unresectable tumor without metastases; age ≥ 18 and ≤ 75 years; Eastern Cooperative Oncology Group (ECOG) performance status (PS) of 0-1; adequate hematological, liver and renal functions. Prior adjuvant chemotherapy and radiotherapy were allowed provided that these interventions had been completed at least 6 months before enrollment in the study.

Major exclusion criteria were: HER2 positive tumor (immunohistochemistry [IHC] 3+ or 2+ with fluorescent *in-situ* hybridization [FISH] amplified); presence of uncontrolled central nervous system (CNS) metastases; prior palliative chemotherapy; pregnancy; breast-feeding; child-bearing potentiality without use of any contraception; any other current or prior malignancy (with the exception of excised cervical carcinoma in situ or squamous cell skin carcinoma), and psychiatric disorders potentially affecting the compliance to the therapeutic program. Patients with clinically significant cardiovascular disease (including myocardial infarction, unstable angina, symptomatic congestive heart failure, serious uncontrolled cardiac arrhythmia) diagnosed ≤ 1 year before enrollment were also excluded.

The study was conducted in accordance with the Helsinki declaration and Good Clinical Practice guidelines; patients provided their written informed consent prior to any study procedure. The protocol was approved by the ethics committees of all participating institutions.

The trial was sponsored by the Italian Oncology Group for Clinical Research (GOIRC). The study was registered at the European Union Drug Regulating Authorities Clinical Trials (EudraCT No. 2009-016962-10).

### Interventions

Upon study entry, a complete medical history was taken, and all the patients underwent a physical examination, evaluation of ECOG PS, laboratory testing including hematology and blood chemistry, computed tomography scan of the abdomen, of the chest, and of all measurable and assessable sites. Bone scan, magnetic resonance imaging scan, and ultrasound endoscopy were carried out only if clinically indicated. Patients subsequently underwent a physical examination and laboratory tests (blood cell count, serum creatinine, bilirubin, AST, ALT) before each cycle of treatment. Tumor evaluations were carried out every 2 months until disease progression or withdrawal from study medication, according to RECIST version 1.1 criteria [15]. In addition, survival was monitored every 2 months in each patient leaving the study. Adverse events were classified according to National Cancer Institute (NCI) common toxicity criteria (CTC), version 3.0.

The dose-dense DCF regimen consisted of docetaxel, 60 mg/m^2^ over a 1-h intravenous (i.v.) infusion on day 1; cisplatin, 50 mg/m^2^ on day 1 (1 to 3- h i.v. infusion); l-folinic acid, 100 mg/m^2^ administered in 5% glucose over 2 h i.v. on days 1 and 2 followed by 5-fluorouracil (5-FU), 400 mg/m2 bolus i.v. on days 1 and 2, and then 5-FU, 600 mg/m2 as a continuous i.v. infusion over 22 h on days 1 and 2. Panitumumab 6 mg/kg was administered intravenously on day 1 before chemotherapy. Pegfilgrastim 6 mg, was given subcutaneously on day 3 at the end of 5-FU infusion.

Patients aged >65 years received a 30% dose reduction of all chemotherapy drugs. Dose of panitumumab was not reduced. Treatment was repeated every 2 weeks and continued up to a maximum of 6 cycles (4 cycles after the first amendment on February 27^th^2012). Maintenance therapy with panitumumab as a single agent was administered until disease progression, unacceptable toxicity, patient’s refusal or physician’s choice. Treatment was delayed in case of insufficient hematological function (neutrophil count <1,500/mm^3^ and/or platelet count <100,000/mm^3^) and/or non-hematological toxicity grade >1 on day 15 of any cycle. No maximum delay was defined in the protocol, but after 3 weeks’ delay, discontinuation of the treatment was left at the investigator’s discretion. In the event of febrile neutropenia, grade 4 non-febrile neutropenia lasting longer than 5 days, or grade 4 or grade 3 with bleeding thrombocytopenia, the dose of each drug was reduced by 25%. The same dose reduction was indicated for grade 3 and 4 non-hematological toxicity (20% in case of panitumumab related skin toxicity, up to a maximum of 60% of its original dose). As for chemotherapy, only one dose reduction was permitted. Delays of panitumumab administration beyond 6 weeks from the previous dose were not allowed.

### Objectives of the study

The primary objective of the study was to evaluate the antitumor activity of panitumumab in combination with a dose-dense chemotherapy regimen in terms of overall response rate (ORR), defined as complete response (CR) and partial response (PR) rates according to RECIST. Further secondary endpoints were safety profile, time to progression (TTP), and overall survival (OS).

### Sample size and statistical methods

The estimate ORR for the treatment with chemotherapy alone was 45% (Dalla Chiesa M et al, ASCO Proceedings 2007). We chose the lower activity (p0) of 0.45. The target activity level (p1) was 0.65. A total of 48 assessable patients were needed to guarantee 80% power under an [alpha]-level of 5%. Assuming that about 10% of patients would have been lost before evaluation (refusal or suspension for toxicity) the number of patients needed to enroll was 52. The ORR was calculated as proportion of patients with the best confirmed response (complete plus partial responses) recorded from the start of treatment until disease progression. Continuous variables were summarized by descriptive statistics. Categorical variables were summarized using counts of patients and percentages. Survival curves for OS and TTP, medians and their 95% confidence intervals were estimated applying the Kaplan–Meier (K–M) method. All subjects enrolled were considered for the Intention to Treat Analysis (ITT). The statistical testing was conducted at the two-sided α=0.05 and 95% confidence interval was employed.

### EGFR expression evaluation

Patients were asked to sign a specific informed consent to donate biological samples (tissue and/or blood) for correlative translational research studies. A small amount of formalin-fixed paraffin-embedded (FFPE) tissue from each block and/or a whole blood sample was therefore retained. The study pathologist assessed the tumor content for each case in one hematoxylin-eosin stained tissue slide. Four 4 μm-thick and eight 8 μm-thick representative serial sections from each pre-treatment biopsy or surgical resections were used for detecting EGFR copy number variation (CNV) by digital droplet polymerase chain reaction (ddPCR). A FISH analysis for confirmation was performed on five samples found to be PCR-amplified.

### Digital droplet PCR

EGFR copy number variation was assessed using ddPCR. The EGFR CNV of the patient samples were determined with a commercial available assay (BioRad, Berkeley, California, USA). This assay was used in parallel (so called duplex PCR-assay) with the assay for the housekeeping gene RPP30 (BioRad, Berkeley, California, USA) that is known to have two copies per diploid genome. All ddPCR analyses were carried out at the Centre for Molecular Pathology of the Institute for Cancer Research (ICR), London, UK. The cut-off for designation of EGFR copy number gain was 2.5.

### Fluorescence *in-situ* hybridization

A Zytolight Spec EGFR/CEN 7 Dual Color Probe (Zytovision, Bremerhaven, Germany) was used to perform FISH. All FISH analyses were carried out at the Laboratory of Molecular Biology Unit of Azienda Socio-Sanitaria Territoriale Hospital of Cremona, Italy.
